# The history of varicocele: from antiquity to the modern ERA

**DOI:** 10.1590/S1677-5538.IBJU.2017.0386

**Published:** 2018

**Authors:** Antonio Marte

**Affiliations:** 1 Unità di Chirurgia Pediatrica, Università della Campania - Luigi Vanvitelli, Napoli, Italia

**Keywords:** Varicocele, history of medicine

## Abstract

Men have most likely been affected by varicocele since the assumption of the upright position. In De Medicina, written during the first century AD, Celsus credits the Greeks with the first description of a varicocele, and he recorded his own acute observation: “The veins are swollen and twisted over the testicle, which becomes smaller”. Celsus himself is credited with the distinction between varicocele (dilation of surface veins) and “cirsocele” (dilation of deep veins). There has been a long history of treatment attempts and failures, some of which are remarkably strange, that have sometimes cul- minated in tragedy, as in the case of French professor Jacques-Mathieu Delpech (1772-1832). Although some questions regarding the etiopathology and treatment of varico- cele remain unanswered, a succession of more or less conservative attempts involving all medical cultures has been performed throughout history. The report by W.S. Tulloch in 1952 brought varicocele into the era of modern evidence-based medicine, and varicocele surgery finally progressed beyond the aim of merely relieving scrotal pain and swelling. From 1970 to 2000, varicocelectomies gained worldwide attention for the treatment of male infertility. Several innovative procedures to correct varicoceles began to appear in the world's literature as interventional radiology, microsurgery, laparoscopy, and robotics, while comprehensive review articles were also published on the subject of varicocelectomies. Microsurgery is nowadays used worldwide and it can be considered to be the gold standard for correcting infertility linked to varicocele.

## ANTIQUITY

Although men have probably been affected by varicocele since the assumption of the upright position, there is no record of this disease in ancient times. In ancient Egyptian medicine, while hernia and hydrocele are well-described in ancient papyri, there is no mention of varicocele, although it was presumably detected frequently during procedures. Several tomb paintings and reliefs depict servants and workmen with protuberances that resemble scrotal swellings (1, 2).

**Figure 1:**
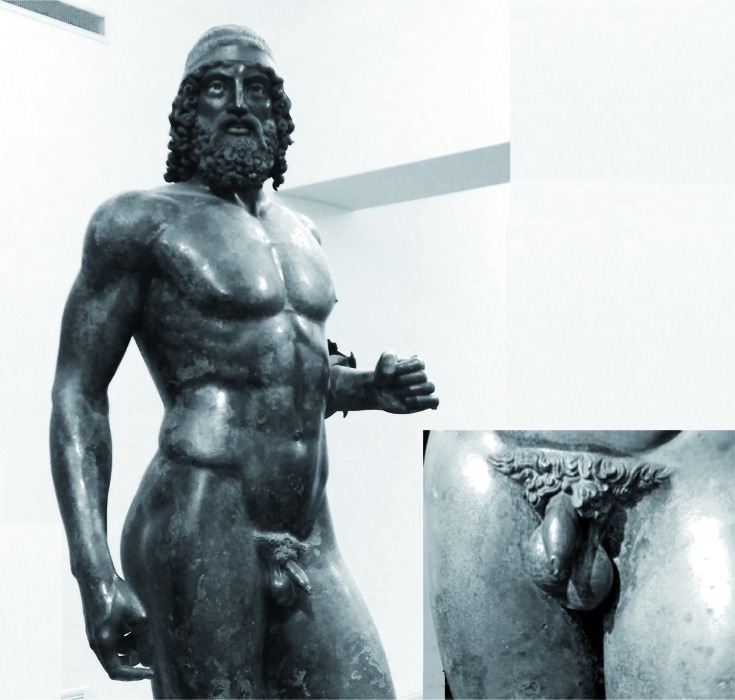
Riace Bronzes: Statue A, the Younger, and detail of the scrotum.

According to a recent study, an illustrious example of ancient Greek art, the famous Statue A- the Younger (Riace Bronzes, fifth century BC- Reggio Calabria, Italy), was modeled with a reproduction of the left varicocele, which the model was probably suffering from at the time (Figure-1) (3).

### The 1st century AD

Varicocele surgery dates back to the first century A.D. According to Hotchkiss, Celsus per-formed the first documented ligation and cauterization of a varicocele (4). Cornelius Celsus (ca. 25BC-ca. 50AD), a Roman nobleman, wrote a general encyclopedia (De Artibus) that covered several subjects, some of which had medical content (De Medicina). It was an eight-volume compendium, including two books on surgery (volumes VII+VIII), and it is the most significant medical document following the Hippocratic writings. Celsus adopted most of the Hippocratic theories and advanced them by presenting a complete description of the etiology, clinical manifestations, and treatments of all diseases and illnesses known at that time. Although largely forgotten for several centuries, Celsus was the first classical medical writer to appear in print (AD 1478) and his writings were highly valued during the Renaissance. Celsus in essence founded the subject of andrology, “ante litteram”, as andrological topics are covered in his “De Medicina”. In De Medicina, written during the first century AD, Celsus credits the Greeks with the first description of a varicocele, and he recorded his own acute observation: “The veins are swollen and twisted over the testicle, which becomes smaller than its fellow”. Celsus himself is credited with being the first to make the distinction between varicocele (dilation of surface veins) and “cirsocele” (dilation of deep veins). Indeed, Celsus warned that the Greek surgeons often con-fused these two varieties, which they both referred to as “kirsokele”;and he added (in De Medicina, VII),“eaeque intortae conglomerataeque venae a superiore parte vel ipsum scrotum implent, vel mediam tunicam, vel imam”, referring to internal dilatation of testicular veins.

**Figure 2:**
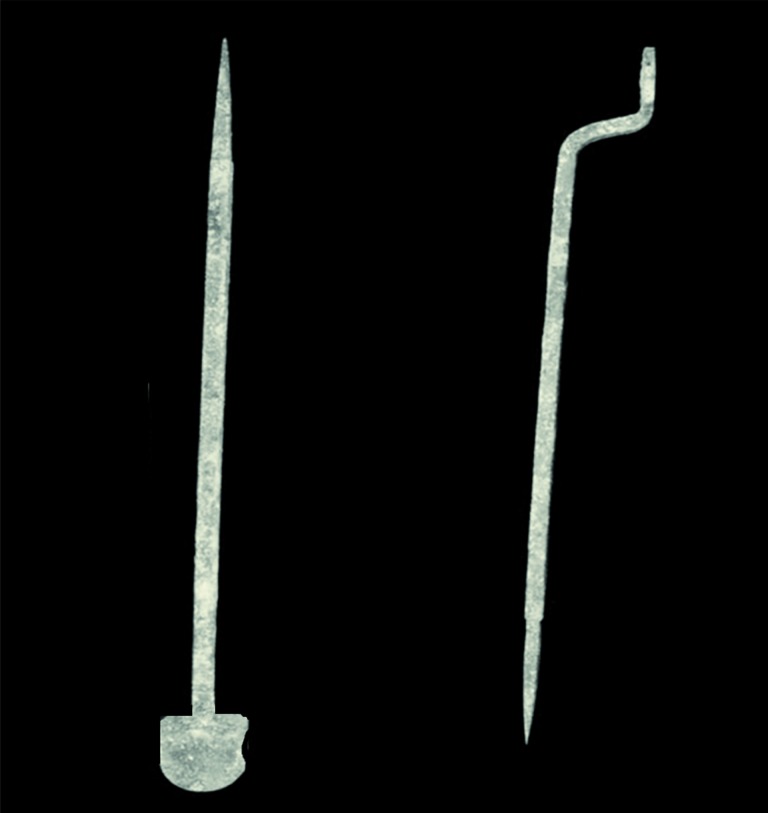
Two cauteries from the 1st century AD. A cautery is a short square-shaped handle with a long, thin or round pointed rod. Roman doctors used cauteries as a counter-irritant, haemostatic, bloodless knife, or as a tool to destroy tumors.

“De Medicina” acknowledges three levels of intervention: for scrotal varicocele, the surgeon should use the cautery (Figure-2); in more severe cases, ligatures are suggested; and if the varicose vein involves the internal lining of the testicle, then removal of the testis is recommended as it has then become completely useless” (5–8). At that time in history, surgeons used herbal mixtures such as opium, mandrake, henbane, and/or hemlock steeped into a soporific or sleep-bearing sponge (“spongia somnifera”). The sponge was dampened so that anesthetic vapors or drippings could be applied to the patient's nostrils. These sponges were likely historical cousins to the so-called Roman or Arabic sponges (used during crucifixions, surgeries, and other painful events) and the most common sutures were made of horsehair or boar bristles (9, 10).

After Celsus, surgical procedures to treat varicocele were performed only via a scrotal approach.

Claudius Galen (130-ca. 200AD) also used the term “cirsocele”, although his description of this condition is rather vague despite his copious scientific production (3). Galen performed surgical resection of the surface scrotal veins by lifting them with a hook before isolating and sectioning them (11).

### The 7th century AD

After the fall of the Western Roman Empire during the Byzantine period, one of the most authoritative medical scientists was Paul of Aegina (625-690AD). Adhering to an encyclopedic approach to medical writing, Paul composed seven books of comprehensive medical knowledge, “Epitomoe Medicoe LibriSeptem”, in which he summarized all of the knowledge available at that time regarding the preservation of health (12).

The sixth book was devoted to surgery. As a highly experienced surgeon, he relied on the prior experiences of Greek and Roman medicine, although he also devised and applied new surgical techniques. In the chapter “On the Excision of Varices”, he wrote: “The varix is a dilatation of a vein occurring sometimes in the temples, sometimes in the hypogastric region below the navel, sometimes in the testicles” Paul was the first to recommend a scrotal approach with isolation and protection of the vas deferens before making the incision above the vascular bundle. Unlike Galen, he recommended ligature of the vein distally and proximally, cutting longitudinally and leaving it open to allow the dumping of clots and secretions.

### The 10th century AD

Albucasis, from Cordoba (936-1013AD), also recommended a scrotal approach to varicocele, and he left a detailed description of the procedure.

“You must have the patient sitting up in a high chair and take hold of the skin of the testicles with your fingers, together with the blood vessels. Make an oblique incision in the direction of the vessels so that they are laid bare. Then run a double threaded needle through them and tie at the spot where the varix begins. Tie it again where the varix ends, then cut through the varix in the middle and draw out the turbid corrupt humidity that has gathered in it. If all of the vessels are varicose, then you will have to remove one testicle for it will be of no use” (13).

Bruno da Longobucco (ca. 1200 Longo-bucco-Padua 1286), in his “Chirurgia Magna” (12,53) states: “It may happen that the skin of the testicles just relaxes and hangs down so horrendously incise the skin and unite the lips of the wound with a suture”. As a result of his extensive knowledge of Greek, Latin, and Arab medicine, Bruno da Longobucco was considered to be one of the greatest surgeons of his time. He was a follower of the surgical practices of Democedes of Crotone, Philistion of Locri, Albucasis, and many others, whose techniques and teaching texts were preserved in Basilian and Benedictine monasteries (14–16). In the centuries that followed, medieval surgery did not contribute much to the treatment of varicocele. Rather, it merely followed the principles outlined by previous authorities.

### The 16th century

**Figure 3:**
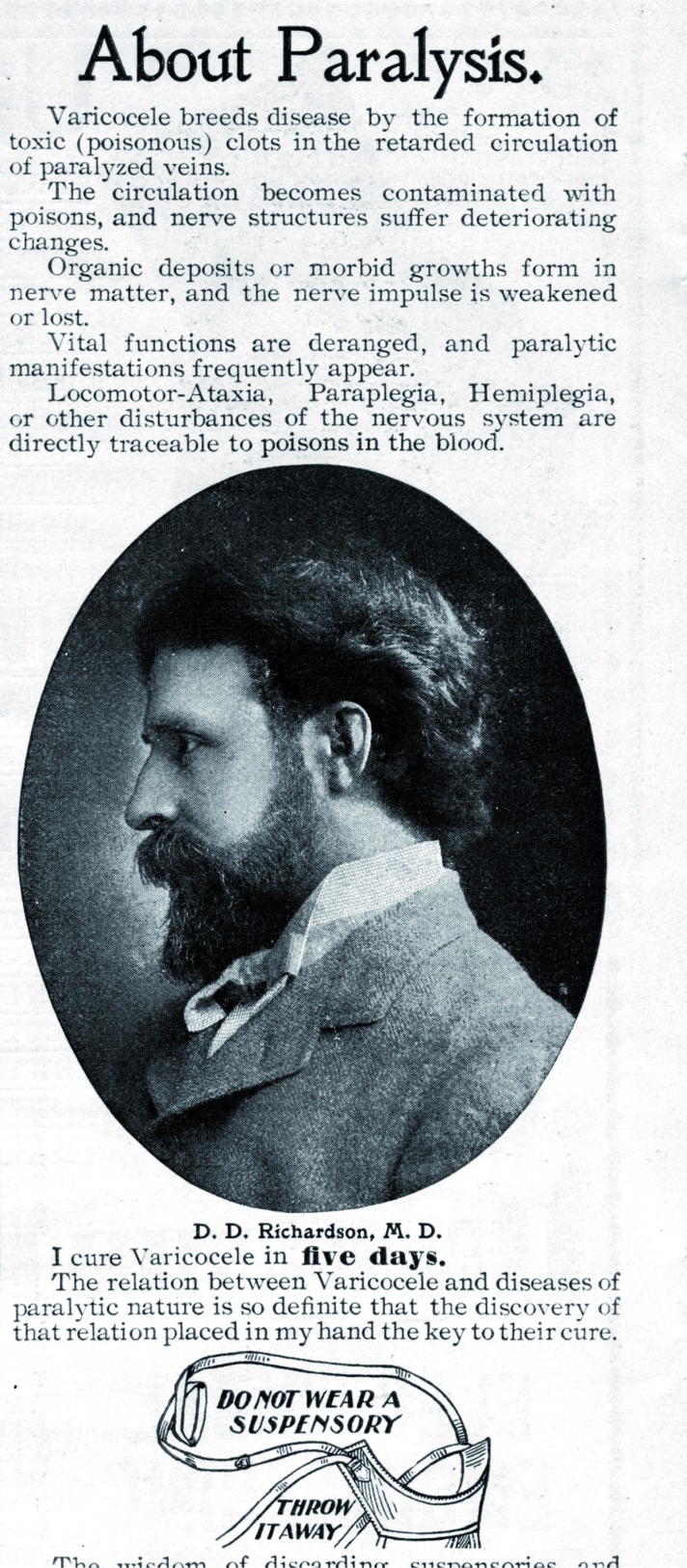
An advertisement in the Chicago Tribune in 1899 heralding the virtues of a varicocele cure in five days. The advertisement states “Do not wear a suspensory. Throw it away”.

In 1541, Ambroise Paré gave the most poetic and effective definition of varicocele. He described a condition of “compact groups of vessels filled with melancholic blood and often growing in men of melancholy temper”. “Melancholic” probably refers to slow and “toxic” blood and one can therefore assume that Paré was aware of blood stasis in varicocele veins. This concept was to remain constant throughout history, and it was resumed in the early twentieth century in the US by some alternative practitioners (Figure-3).

Paré recommended delivery of the dilated veins through a 2-inch scrotal incision with subsequent use of a double ligature instead of cautery (17).

The great French surgeon and anatomist Pierre Dionis (1643-1718) had a more conservative attitude.

“If there is a varicocele (considered as a dilation of the surface veins of the scrotum), it is necessary to start by prescribing numerous bleeds to drain the vessels, and to impose a correct lifestyle to prevent the vessels from filling up again; then apply to the swollen part a large compress soaked in astringent wine and cover it with a sus-pensor to support and compress the areas to facilitate the correct outflow of the blood. In ancient times these veins were cauterized in several places with pointed and rounded cauteries, but this excessively harsh procedure is no longer used today. Nowadays, these veins tend to be opened with an S-shaped scalpel when general remedies like astringent wine and the suspensor fail to offer the patient relief.

The surgeon opens the veins in the areas that are more dilated, draining out all the blood, using astringent wine and the suspensor. This technique will enable healing, ensuring that the new blood can continue to circulate.

If there is a cirsocele (considered to be the veins inside the scrotum), all authors agree that there is only one form of treatment, this being amputation of the testicle: I personally find that the remedy is worse that the ailment, and I have never used this technique.

I recommend bleeding from time to time, a restrictive diet, abstinence from strenuous exercise, and the constant use of a suspensor to provide relief from pain when the testicle is not supported; and unless obligated by severe need, treatment of this disease should not be undertaken at the expense of the testicle” (18, 19).

### The 18th century

Sir Astley Paston Cooper, 1st Baronet (1768-1841), was an English surgeon and anatomist who made historical contributions to otology, vascular surgery, the anatomy and pathology of the mammary glands and testicles, and the pathology and surgery of hernia. Astley Cooper, a student of the eminent John Hunter, became the most acclaimed surgeon of his time. He provided a very personal interpretation of varicocele as “orchidoptosis”, consisting of a plastic reduction of the scrotal sac (Figure-3).There is a famous story told by Cooper about one of his patients, an experienced rider, who after undergoing this procedure traveled 50 miles on horseback without any pain (20, 21).

**Figure 4:**
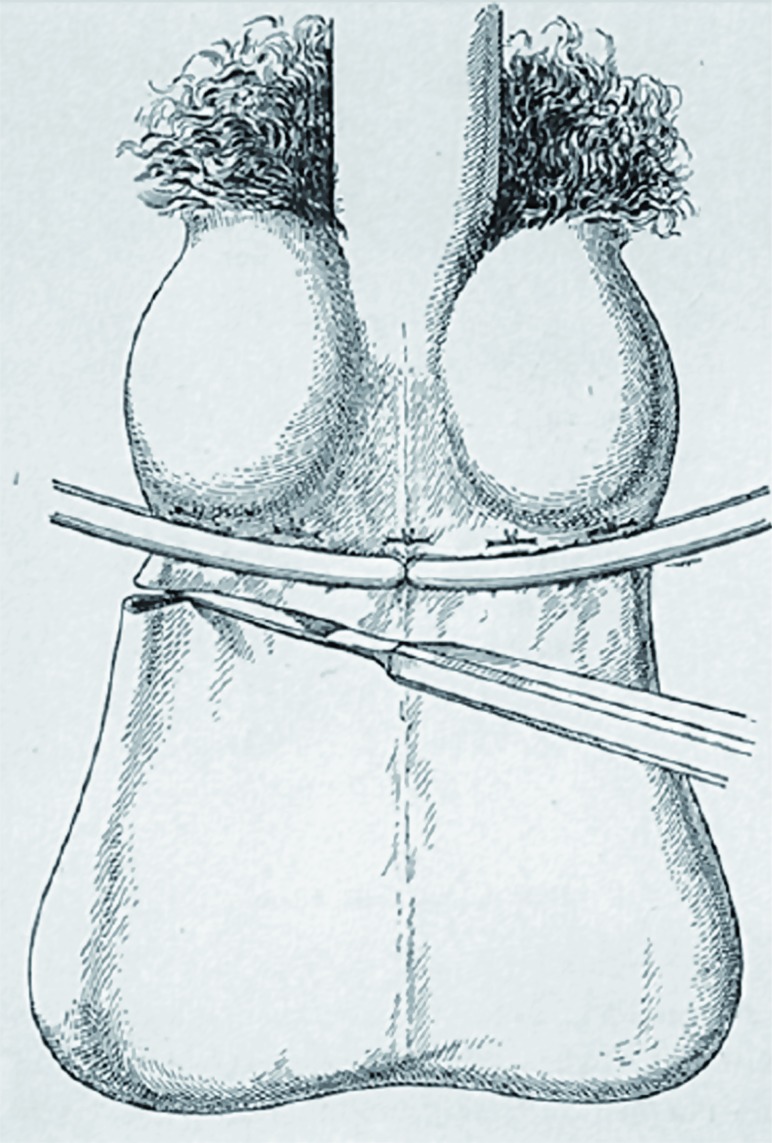
Cooper's reduction scrotoplasty: partial excision of the scrotum, leading to an upward adjustment of the affected testicle (“inner support”).

**Figure 5:**
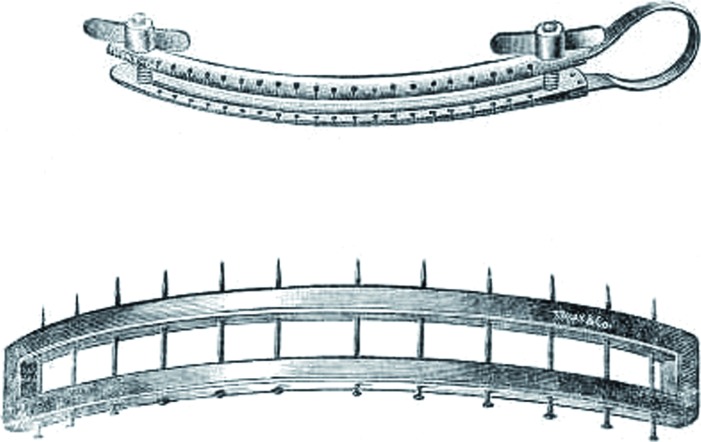
King's (upper) and Andrew's (lower) scrotal clamp. Reduction scrotoplasty, unlike that of cooper, involved the application of the clamps longitudinally following the median raphe. Andrew's clamp was specifically designed to obviate injurious pressure on the tissue during the operation.

**Figure 6:**
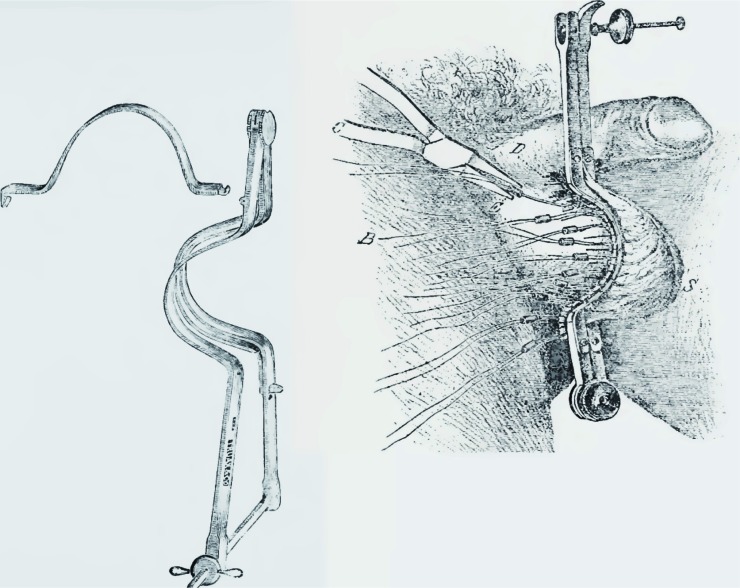
Heurteloup's scrotal clamp. It was an effective cutting device to isolate and cut the redundant scrotum. After having detached the removable blades, transfixed wires blocked with lead beads were applied.

From then on, a wide range of devices for scrotal section was developed: Heurteloup, King, and the Lewis scrotal clamps (Figures 4–6). At the beginning of the 19th century, the most common form of intervention was a double-thread ligature (silver, lead) of the entangled veins at the base of the scrotum, sparing the deferent and the deferential artery. Special needles were used for the passage (22).

**Figure 7:**
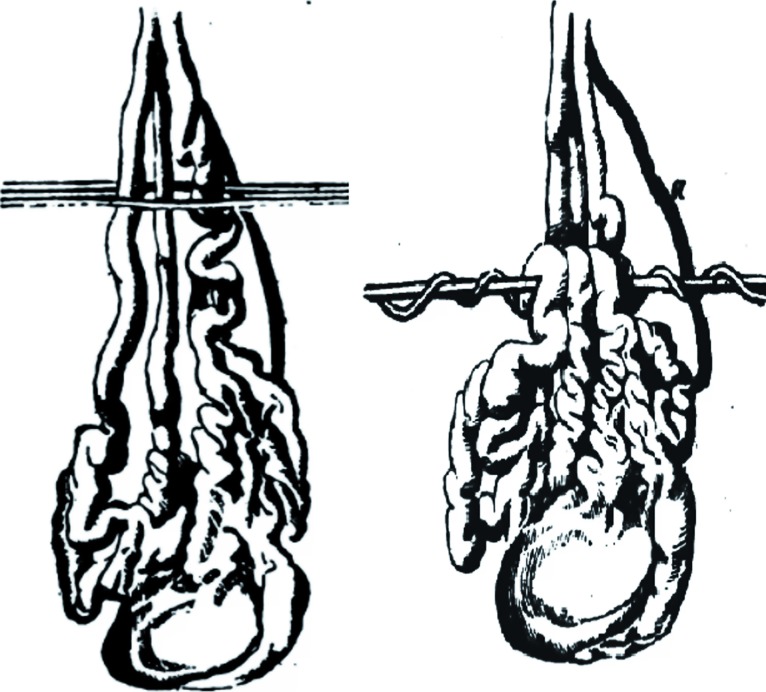
Vidal's technique: with the patient standing upright, the vas deferens were isolated and two silver wires, one large and the other one small (i.e. one thicker, the other one thinner), were passed behind and in front of varicose veins. The two wires were then progressively rolled until the veins ulcerated.

One of the earliest surgical procedures was that of Vidal de Cassis. This method consisted of passing an iron pin through the scrotum between the vas deferens and the enlarged veins. A silver wire was then passed along the pin outside the veins, which were included between the pin and the wire. The wire was then fastened to the ends of the pin, and the pin was twisted to exert a certain amount of pressure on the vessels. The twisting process was repeated every day or every other day until the veins ulcerated and the pin became loose; both the pin and the wire were then withdrawn. The veins were sectioned and inflammatory adhesions were eliminated (Figure-7) (23).

**Figure 8:**
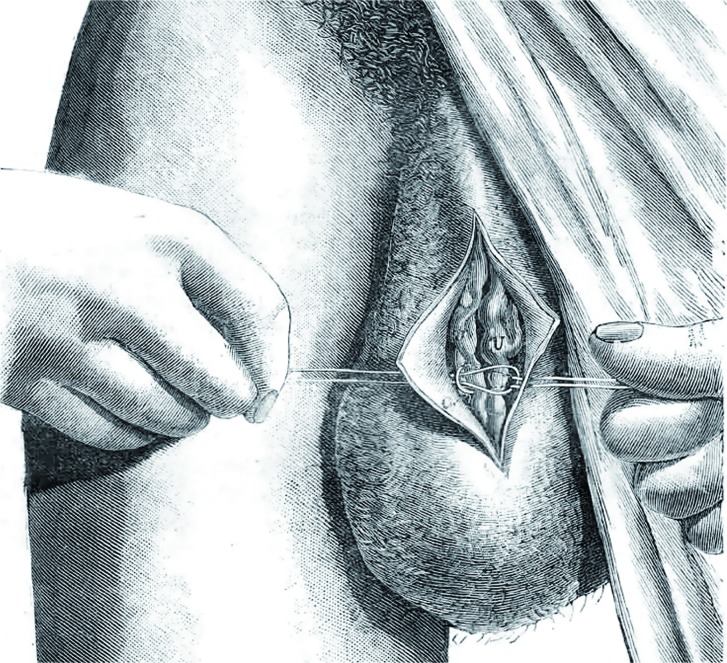
One of the most popular techniques for varicocele ligation in the 19th century (scrotal window) involved ligature of the varicose packet with two loops, anterior and posterior, passed with a transfixed needle, thereby saving the vas deferens.

The technique involving ligature at the base of the scrotum was also used by the French surgeon Jacques Mathieu Delpech (Toulouse 1772-Montpellier 1832), who, in some cases, alternated it with a type of sclerotizing treatment: after longitudinally cutting into the scrotum and exposing the venous plexus, he applied a coating of Touchwood mushroom (a cauterizing agent for wounds, already described by Hippocrates in the 5thcentury BC), which was removed after four days (24) (Figure-8).

The simultaneous treatment of a bilateral varicocele, which was a remarkable feat for that time, resulted in the death of the great French surgeon, who was murdered by one of his patients, Marc Demptos, who he was said to have operated on unsuccessfully a year earlier for bilateral varicoceles. The story was in fact considerably more complex than that. Delpech was murdered because he was accused of having betrayed professional secrecy. On Sunday, October 28th, 1832, during an evening at the theatre with his son, Delpech was approached by Demptos, who asked the Professor to retract the news given to the mother of his wealthy fiancée about his infertility as a result of the operation. They had a very heated discussion and Demptos left the theatre highly enraged. On Monday, October 29th, Delpech left Saint-Éloi Hospital (presently the Rectorate of Montpellier) for the Institute of Orthomorphia (modern-day Orthopedics). He was accompanied by his valet who drove the carriage. As the carriage reached the road to Toulouse, Demptos came out of a house holding a double-barrel rifle. He shot twice, killing Delpech and his valet on the spot, after which he returned to his room and shot himself.

The autopsy revealed the murderer's testes to be soft and shrunken, presumably from the operation (25, 26).

For centuries, varicocele was treated solely in order to relieve the dragging weight and pain. The 19th century literature is full of various treatment methods recommended for pain relief, for when the testicle was atrophic, or in case the sufferer had been disqualified from public service. In the mid-19th century (1856), Thomas Blizard Curling (London 1811-1888), observed “a decrease in the secreting powers of the gland” and suggested, for the first time, a relationship between varicocele and male infertility. Curling's name is also linked to the definitive adoption of the term varicocele rather than cirsocele, which was originally coined in 1843 to describe the pathologic dilatation of veins of the spermatic cord and the procedure for diagnosing varicocele by reducing the swelling with the patient in a supine position and then palpation in an upright position (27).

### The 19th century

The end of the 19th century was a time of “radical cures” for many common surgical diseases. Eduardo Bassini's “radical operation of the inguinal hernia” was developed and perfected in 1883-1887. In 1890, Bassini published successful results in regard to 262 hernia repairs. This new technique not only changed the approach to hernia repair, but also to inguinal surgery in general. Bassini's contribution was to focus on the rear wall as the real repair site by approximating the internal oblique muscle, the transversus abdominis muscle, and the transversalis fascia with the iliopubic tract and the shelving edge of the inguinal ligament (28).

Dr. Albert Narath (1864-1924), a professor of surgery in Utrecht and then in Heidelberg (1906-1910), had broad surgical interests, including the treatment of varicocele. In 1900, he described the first truly inguinal approach to dilated spermatic veins. The idea of using Bassini's procedure “to section the main trunk of the vena spermatica interna in the inguinal canal” came from the recognition of “venec- tasia” within the inguinal canal during hernia repair. Narath first performed this procedure in 1898. Two years later, Narath concluded that “these inguinal incisions are undoubtedly preferable to the old scrotal incisions”. This operation was the first to shift the focus from the scrotum to the inguinal area (29).

### The 20th century

After these events, varicocele surgery was shaped largely by developments in Central and South America. In his report in 2014, Gonzalez laid the foundation for a proper designation of surgical procedures that, by eponyms, are identified using the names of these surgeons (30). This distinction plays a very important role because, at present, these procedures are still among the most popular surgical techniques for this condition. In 1918, Dr. Oscar Ivanissevich, working in Buenos Aires, described the anatomy of the spermatic vein, and he proposed a suprainguinal approach to spermatic vein ligation. The rationale for this approach was to ligate the vein where it was most likely to have a single trunk. In 1960, he reported his experience with more than 4.000 cases using the suprainguinal approach and he provided detailed illustrations regarding his technique. Bernardi, a disciple of Ivanissevich, advocated a transinguinal approach to spermatic vein ligation. In his 1960 article, however, Ivanissevich is critical of Bernardi's transinguinal approach, stating that this approach is more likely to encounter multiple venous trunks and risk missing veins. In 1949, Palomo described a procedure that involved the ligation of artery and vein, without the risk of testicular atrophy, in the retroperitoneal space. This is the procedure that is used nowadays, both in open and laparoscopic surgeries.

Doctor Alejandro Palomo was a urologist who was born in Guatemala in 1917. He graduated from the San Carlos University Medical School of Guatemala in 1942. He trained in Urology at New York Hospital, under Dr. Oswald Lowsley, from 1943 to 1945, and subsequently returned to Guatemala. In Guatemala, he was Professor of Urology at San Carlos Medical School and he served as Chief Urologist at Guatemala City Hospital, where he developed his technique for the treatment of varicocele. In a 1947 publication entitled “Radical cure for varicocele. Modification of Doctor Ricardo Bernardi's technique”, he described ligation of both spermatic arteries and veins at the internal inguinal ring. The classical report was then published in 1949 as “Radical Cure of a Varicocele by a New Technique” (31). Based on a study of a small group of 40 men, he noted that three arteries supplied the testis. He concluded that, as long as only two arteries were ligated, the flow from the remaining artery would supply sufficient blood to the testis.

The procedure was carried out under local anesthesia. The incision for this procedure was 4cm in length and 3cm above the internal ring. The dissection was just above the internal inguinal ring, where the large spermatic veins are readily visible. Although the artery and veins were ligated together, Palomo excluded the deferential and cremasteric arteries, which he believed supplied sufficient blood to the testis. Among his first 40 cases, there were no relapses or evidence of atrophy, although hydrocele formation was not discussed.

Some authors have mistakenly applied the term “modified Palomo” procedure to the ret-roperitoneal approach preserving the artery, as it is in fact the Ivanissevich procedure (32–35).

The first studies to document an improvement in semen quality and an increase in pregnancy rates following treatment of varicocele were by Barwell in 1885, Bennett in 1889, and Macomber and Sanders in 1929 (36–38).

William Selby Tulloch (1913-1988) was the first surgeon to repair a varicocele for the treatment of infertility. His initial report described an infertile man with bilateral varicoceles and testicular biopsy-proven maturation arrest. This patient was able to attain an increase in sperm concentration and give rise to a natural pregnancy after their varicocele was repaired. Tulloch used the Robb procedure, which approached the spermatic veins 5 cm above the internal inguinal ring. At this site, he felt the dilated veins were fewer in number and the arterial blood supply to the testis could be avoided (39, 40).

Tulloch's report contributed to the worldwide acceptance of the role of varicocele in male infertility. With these new aims, varicocele surgery entered the modern age, making use of the increasingly sophisticated technologies that were becoming available.

This brings us to the field of operative radiology, the use of the operating microscope, laparoscope, and robot-assisted laparoscopy.

In 1976 Comhaire and Kunnen (41) demonstrated that when contrast medium is injected during selective retrograde catheterization of the left internal spermatic vein at its orifice in the renal vein, patients with varicocele, standing in the erect position, present a retrograde filling of the varicose spermatic vein. Later, in 1978, Lima et al. induced the sclerosis of re-fluxing veins by catheterization of the internal spermatic veins with the injection of a 75% hypertonic glucose solution (42). The injection was repeated several times until the caliber of the vein was significantly reduced. In light of these results, other substances and devices have been tested, such as 2-isobutyl-cyano acrylate, steel coils, and detachable balloons (43). Although the development of sclerosing techniques were originated in the 20st century, the development continued into the 21st century. Further progress towards achieving effective and minimally invasive treatment of vein reflux was made by Tauber, who introduced sclerotization via direct injection of a sclerotizing substance through a cannula into a refluxing vein. The procedure could be carried out on an outpatient basis and under local anesthetic through a small incision at the root of the hemiscrotum, distal ligation (to avoid accidental injection toward the testis), and injection of 3mL of sodium morrhuate with the “air block technique” (44). The technique was then described in detail in the “Surgery Illustrated” published by the British Journal of Urology in 2006, presenting a case history of over 6.000 patients and using the less toxic polidocanol at 3% instead of sodium morrhuate (45).

**Figure 9:**
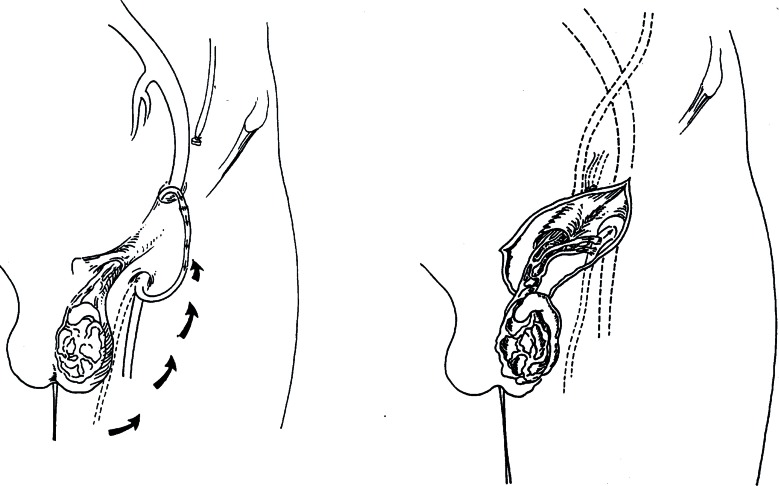
Microsurgical anastomoses. (Left) Ishigami's technique: testicular-saphenous anastomosis. (Right) The FOX technique: direct anastomosis of two or three dilated veins of the pampiniform plexus with the great saphenous vein.

The advent of the operating microscope and the affirmation of microsurgery marked another important step forward in the treatment of varicocele. In fact, the operating microscope was first used at the beginning of the 1970's, with various forms of vascular microanastomosis techniques, with the intention not to completely close the refluent veins, but to create venous outflux into another vascular area. Ishigami was the first to propose terminal-terminal microanastomosis between the spermatic vein and the saphenous vein (Figure-9). This operation presented certain negative elements in that, aside from requiring two incisions, one on the thigh and one at inguinal level, it created a risk of stenosis or thrombosis of the anastomosis due to the long subcutaneous tunnel required by the transposition of the saphenous vein (46).

Further microanastomosis techniques followed: terminal-lateral between the spermatic and saphenous veins, and between the spermatic vein and the distal portion of the lower epigastric vein (47, 48). However, micro-surgical diversions proved to be too complex for routine usage (49). In addition, there were other complications related to varicocele surgery such as injuries to the testicular arteries (50) and disruption of the lymphatics that produced post op hydroceles (51).

**Figure 10:**
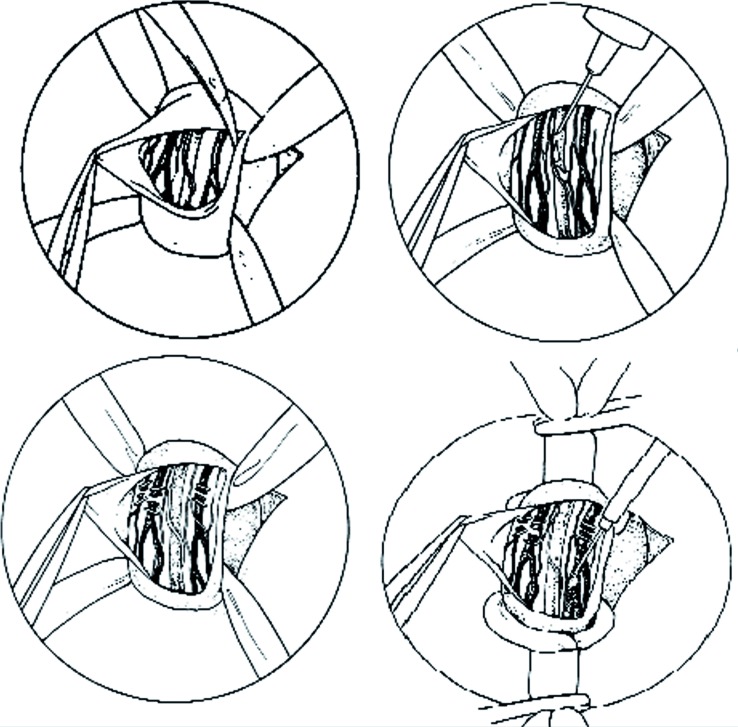
Marmar's subinguinal microsurgical technique. Delivery of the testis is not performed; the varicose veins are clipped with hemoclips and transected, with controlled sclerosis of small cross-collateral veins. Proximal and distal control of the spermatic cord is obtained by cinching the Penrose drains.

In 1985, Marmar et al. (52) proposed a combined microdissection of the spermatic cord at the external inguinal ring, ligation of the dilated veins, and controlled sclerosis of small cross-collateral veins. The procedure was performed with an operating microscope and microsurgical instruments. Among 71 cases, there were no post hydroceles and 2 palpable recurrences (0.28%). In this initial report, the semen parameters demonstrated statistically significant improvement and the pregnancy rate was 29.9%. However, in 1994, Marmar and Kim reviewed their experience with 466 subinguinal microsurgical varicocelectomies. There was only 1 permanent hydrocele, a palpable recurrence rate of 0.82% and a 1yr pregnancy rate of 35.6% (53) (Figure-10).

**Figure 11:**
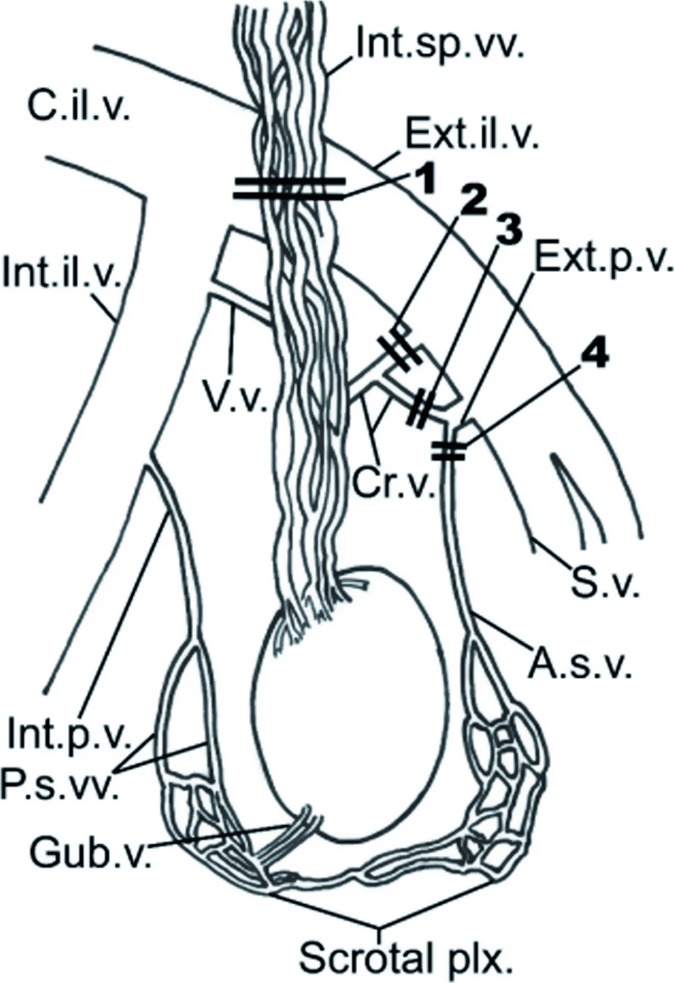
Goldstein's subinguinal microsurgical technique. This technique involves a more aggressive approach with arterial dissection and venous ligation, and delivery of the testis as part of the procedure to ligate gubernacular veins.

In 1992, Goldstein modified the micro-surgical, subinguinal varicocelectomy, taking a more aggressive approach with arterial and lymphatic microsurgical dissection and venous ligation by an arterial and lymphatic sparing technique that in most procedures involved delivery of the testis (54). The authors reported a failure rate of 0.6% for all of the procedures, and a pregnancy rate per couple of 43% within 6 months (Figure-11). In time, other investigators questioned the need to deliver the testicle as part of a microsurgical inguinal varicocelectomy, for example Ramasamy and Schlegel (55) in their comparative study, demonstrated that there were no varicocele recurrences with either procedure, and that delivery of the testis did not offer any beneficial effects on semen quality or pregnancy rates after varicocelectomy. Microsurgery is nowadays used worldwide and it can be considered to be the gold standard for correcting infertility linked to varicocele.

Cayal et al. encountered significant differences among the techniques as they found overall natural pregnancy rates of 37.69% for the Palomo technique series, 41.97% for micro-surgical varicocelectomy techniques, 30.07% for the laparoscopic varicocelectomy techniques, 33.2% for radiologic embolization, and 36% for the macroscopic inguinal (Ivanissevich) varicocelectomy series (p=0.001) (56, 57). The authors concluded that open microsurgical or subinguinal varicocelectomy techniques have been shown to result in higher pregnancy rates, fewer recurrences and postoperative complications than conventional varicocelectomy techniques in infertile men. Use of higher magnification allows surgeons to preserve the testicular arteries and lymphatics and also to visualize and occlude all spermatic veins. However, further prospective randomized trials are needed to directly compare with other treatment modalities in infertile men with varicoceles. A more recent report by Shulster et al. regarding simultaneous treatment of varicocele and inguinal hernia showed that microsurgical techniques can also minimize the complications of inguinal hernia repair, such as vasal obstruction, testicular atrophy, recurrence, infection, hematoma, chronic postoperative pain, and loss of sensation (58).

In the mid-1980s, the introduction of laparoscopic cholecystectomy represented a historical turning point that was as momentous as the discovery of anesthesia, asepsis, antibiotics, extracorporeal circulation, and the use of operating microscopes (59). In 1991, Aaberg reported the first experiences of Palomo performed using laparoscopy (60). In 1992, Hagood et al. and Donovan et al. (61, 62) reported laparoscopic varicocelectomies with sparing of the spermatic artery. They reported that a laparoscopic camera provided a good level of magnification of vascular structures. The arteries could readily be visualized after a papaverine drip, and the internal spermatic veins were identified and clipped without difficulty. Donovan reported a mean operating time of 101/153 minutes.

The principle of laparoscopic varicocele ligation is based on the following steps: peritoneal approach; opening a small window on the posterior peritoneum at a distance of 1-2cm from the inner inguinal ring; isolation of the vessels and their ligation or sealing “en bloc” or exclusion of the artery and the lymphatics. The procedure is facilitated by the magnifying effect of the laparoscopic lens, which allows for excellent visualization of the structures of the vascular bundle.

To achieve better visualization of the lymphatics, recent findings have shown that the intra-dartos/intra-testicular injection of isosulfan blue is significantly better than the previously described intra-dartos injection, thereby allowing for identification of lymphatic vessels in 100% of the cases in our series (63).

### The 21st century

With the increase in familiarity regarding the use of laparoscopy and technical progress through the introduction of curved instruments, the procedure was recently also carried out with SILS (Single Incision Laparoscopic Surgery). In 2008, Kaouk et al. reported their initial experience in children using a multichannel single laparoscopic port inserted in the umbilicus. The testicular vessels were then dissected from the lymphatics, and the vessels-both artery and veins- were transected leaving clips both proximally and distally (64). In 2014, Marte et al. have reported their experience with SILS laparoscopic Palomo varicocelectomy in adolescents compared to the traditional procedure. The results revealed no significant difference in terms of the operating time and the incidence of secondary hydrocele, although the postoperative pain score was significantly better with SILS (65). Another minimally invasive approach is represented by retroperitoneal varicocelectomy. This technique uses one 12mm trocar with a short, 27cm, 0 operative telescope and a 5.5 operating channel. An incision is made right below the 12th rib at the posterior axillary line. A muscle-splitting dissection is performed to gain access to the retroperitoneal space. The port is installed and CO2 insufflation is initiated to create the working space, which is progressively enlarged by moving the type of telescope. Once the retroperitoneal working space is created, the spermatic vessels are identified at the site where they cross the ureter. The testicular artery and one or two veins are dissected from the peritoneum and then coagulated by monopolar or bipolar electrocautery (66).

This brings us to the present day situation and the affirmation of robot-assisted surgery has led to the first published reports of robot-assisted varicocelectomy in both adult and pediatric patients. Corcione et al. were the first to use a robot-assisted da Vinci® platform in association with a laparoscopic varicocelectomy (67). Shu et al. performed the first eight robot-assisted subinguinal varicocelectomies and they compared the data relative to eight patients who had conventional microsurgical procedures. The operating times were the same and neither group experienced complications (68). Hidalgo et al. reported their experience with robot-assisted left-side varicocelectomy in four pediatric patients with a mean age of 15.3 years. The authors reported no significant difference in operative time (p=0.02) and no intraoperative or postoperative complications, although the costs for the robot-assisted group were significantly higher (i.e. $15.800 vs. $8.600, p=0.0005) (69).

In conclusion, it is safe to say that treatment of varicocele has entered the age of modern evidence-based medicine, and that varicocele surgery has finally progressed beyond merely providing relief of scrotal pain and swelling. There is now convincing evidence that varicocele may have a progressive harmful effect on the testes, resulting in a decline in semen parameters. Recent studies on the pathophysiology of varicocele-related infertility have revealed the likely influence of ultrastructural testicular changes and increased oxidative stress, with implications for the seminal antioxidant capacity and sperm chromatin integrity (70). The methods used to correct varicoceles started from crude beginnings. However, in recent years, there have been innovative advances in surgical techniques to correct these lesions. In addition, there has been striking developments of biomolecular and functional sperm tests (71, 72) to evaluate infertile men with varicoceles. Therefore, going forward, it should be possible to better understand the mechanism leading to infertility caused by varicoceles, and the techniques reported in this text will offer effective ways to reverse the problems.
